# Supplementary material for: Regulatory Role of a Hydrophobic Core in the FliG C-Terminal Domain in the Rotary Direction of a Flagellar Motor
Source: Biomolecules. 2025 Feb 1;15(2):212. doi: 10.3390/biom15020212 (PMC11853002; doi:10.3390/biom15020212)
Supplement: Supplementary file 1 [file biomolecules-15-00212-s001.zip › biomolecules-3420369-supplementary.pdf]

biomolecules MDPI

Supplementary Materials

Regulatory Role of a Hydrophobic Core in the FliG C-Terminal Domain in the Rotary Direction of a Flagellar Motor

Nishikino *et al.*

*Corresponding author. E-mail: nishikino.tatsuro@nitech.ac.jp

This PDF file includes:

Figure S1
Table S1

Figure S1. Amino acid sequence alignment of FliG.

```
V.a.  MANDIVPQDDNAAGMPVELDVSTITGEEKAAILLLSLNEQDAAGIIRHLE  50
T.m.  ------------------MPEKKIDGRRKAAVLLVALGPEKAAQVMKHLD  32
S.e.  --------------------MSNLSGTDKSVILLMTIGEDRAAEVFKHLS  30
E.c.  --------------------MSNLTGTDKSVILLMTIGEDRAAEVFKHLS  30
A.a.  ----------------MAQEKSALSKAQKAAVLLLSLPEEVSMNIVKELS  34

V.a.  PKQVQRVGSAMAKAKDLSQDKVSAVHRAFLEDIQKYTNIGMGSEDFMRNA  100
T.m.  EETVEQLVVEIANIGRVTPEEKKQVLEEFLSLAKAKEMISEGGIEYAKKV  82
S.e.  TREVQALSTAMANVRQISNKQLTDVLSEFEQEAEQFAALNINANEYLRSV  80
E.c.  QREVQTLSAAMANVTQISNKQLTDVLAEFEQEAEQFAALNINANDYLRSV  80
A.a.  EEELQKLFALAKDLESVPEEEIENIAEELLDEIKKAGIKIKKPEEFIENI  84

                                              E144D Q147H
V.a.  LVAALGEDKANNLVDQILLG-TGSKGLDSLKWMDPRQVASIIVNEHPQIQ  149
T.m.  LEKAFGPERARKIIERLTSS-LQVKPFSFVRDTDPVQLVNFLQSEHPQTI  131
S.e.  LVKALGEERASSLLEDILETRDTTSGIETLNFMEPQSAADLIRDEHPQII  130
E.c.  LVKALGEERAASLLEDILETRDTASGIETLNFMEPQSAADLIRDEHPQII  130
A.a.  -KKVIPPTLAEKFRGILELG-DAEKILKEIEKVDSRILASLLKNEHPQTI  132

                                        P188L
V.a.  TIVLSYLEADQSAEILSQFPERVRLDLMMRIANLEEVQPSALAELNEIME  199
T.m.  AVVLSYLDPPVAAQILGALPEELQTEVLKRIALLERTSPEVVKEIERNLE  181
S.e.  ATILVHLKRSQAADILALFDERLRHDVMLRIATFGGVQPAALAELTEVLN  180
E.c.  ATILVHLKRAQAADILALFDERLRHDVMLRIATFGGVQPAALAELTEVLN  180
A.a.  ALFLSQLSPKKSAEIIQNLPEELKKEVVKRIATLENVNVQYVKELAQILL  182

             G215A A218V
          G214S
V.a.  KQFAGQAGAQAAKIGGLKAAAEIMNYLDNNVEGLLMEQIRDQDEDMATQI  249
T.m.  KKISGFVSRTFSKVGGIDTAAEIMNNLDRTTEKKIMDKLVQENPELADEI  231
S.e.  GLLDGQ-NLKRSKMGGVRTAAEIINLMKTQQEEAVITAVREFDGELAQKI  229
E.c.  GLLDGQ-NLKRSKMGGVRTAAEIINLMKTQQEEAVITAVREFDGELAQKI  229
A.a.  EEISSLGAKEALKLEGTAVAAELLNTLDKETRELILQSIGQEDPLLEERI  232

      D251R    L259Q     L270P L271R   A282T
V.a.  QDLMFVFENLVEVDDQGIQKLLRDVPQDVLQKALKGADDSLREKVFKNMS  299
T.m.  RRRMFVFEDILKLDDRSIQLVLREVDTRDLALALKGASDELKEKIFKNMS  281
S.e.  IDEMFLFENLVDVDDRSIQRLLQEVDSESLLIALKGAEPPLREKFLRNMS  279
E.c.  IDEMFLFENLVDVDDRSIQRLLQEVDSESLLIALKGAEQPLREKFLRNMS  279
A.a.  REKMFTFEDIRKLSDRDIIEILKVVDKNTLMIALLGAPEDIKQKFLSNMS  282

                                L329Q
V.a.  KRAAEMMRDDIEAMPPVRVADVEAAQKEILAIARRMADAGELMLSGGADE  349
T.m.  KRAAALLKDELEYMGPVRLKDVEEAQQKIINIIRRLEEAGEIVIARGGGE  331
S.e.  QRAADILRDDLANRGPVRLSQVENEQKAILLIVRRLAETGEMVIGSGEDT  329
E.c.  QRAADILRDDLANRGPVRLSQVENEQKAILLIVRRLAETGEMVIGSGEDT  329
A.a.  KRAAKLFLEDMEALGPVKKSEIEKAQRQVVNIIRKMIDEGKIEIGD----  328

V.a.  FL--  351
T.m.  ELIM  335
S.e.  YV--  331
E.c.  YV--  331
A.a.  ----  328
```

The aligned sequences were from *Vibrio alginolyticus* (*V.a.*), *Thermotoga maritima* (*T.m.*), *Salmonella enterica* (*S.e.*), *Escherichia coli* (*E.c.*), and *Aquifex aeolicus* (*A.a.*). Green, yellow

green, red, blue, and black arrowheads indicate mutation sites that increased motor switching, motility defects that were suppressed by the mutation in the CCW state, CW-biased rotation, CCW-biased rotation, and motility defects, respectively. The EHPQR motif, Gly-Gly flexible linker, MFXF motif, and torque helix are highlighted in orange, light green, pink, and khaki, respectively.

Table S1. List of nucleotide sequences of the primer for the *Vibrio* FliG mutations.

Mutation site	Nucleotide sequences
G214S_forward	CAGCCAAGATTAGCGGCCTGAAAGCGG
G214S_reverse	CCGCTTTCAGGCCGCTAATCTTGGCTG
G215A_forward	CAAGATTGGCGCACTGAAAGCGGCAG
G214S_forward	CTGCCGCTTTCAGTGCGCCAATCTTG
D251R_forward	TGGCGACGCAAATTCAACGTTTGATGTTTGTCTTC
D251R_reverse	GAAGACAAACATCAAACGTTGAATTTGCGTCGCCA
M253I_forward	GACGCAAATTCAAGACTTGATTTTTGTCTTCGAAAACTTAG
M253I_reverse	CTAAGTTTTCGAAGACAAAAATCAAGTCTTGAATTTGCGTC
V255G_forward	CAAGACTTGATGTTTGGTTTCGAAAACTTAGTCGAAG
V255G_reverse	CTTCGACTAAGTTTTCGAAACCAAACATCAAGTCTTG
V255W_forward	CAAGACTTGATGTTTTGGTTCGAAAACTTAGTC
V255W_reverse	GACTAAGTTTTCGAACCAAAACATCAAGTCTTG
F256A_forward	GACTTGATGTTTGTCGCGGAAAACTTAGTCGAA
F256A_reverse	TTCGACTAAGTTTTCCGCGACAAACATCAAGTC
E257K_forward	GATGTTTGTCTTCAAGAACTTAGTCGAAGTGGAC
E257K_reverse	GTCCACTTCGACTAAGTTCTTGAAGACAAACATC
L259Q_forward	TTGTCTTCGAAAACCAAGTCGAAGTGGACG
L259Q_reverse	CGTCCACTTCGACTTGGTTTTCGAAGACAA
L270P_forward	AAGGTATTCAGAAAAGGCTGCGTGATGTGC
L270P_reverse	GCACATCACGCAGCCTTTTCTGAATACCTT
L271R_forward	TATTCAGAAATTGCCGCGTGATGTGCCAC
L271R_reverse	GTGGCACATCACGCGGCAATTTCTGAATA
Q325F_forward	GATGTCGAAGCGGCATTCAAAGAAATCCTAGC
Q325F_reverse	GCTAGGATTTCTTTGAATGCCGCTTCGACATC
L329Q_forward	GGCACAGAAAGAAATCCAAGCGATCGCTCGTC
L329Q_reverse	GACGAGCGATCGCTTGGATTTCTTTCTGTGCC
A332F_forward	GAAATCCTAGCGATCTTCCGTCGCATGGCCGATG
A332F_reverse	CATCGGCCATGCGACGGAAGATCGCTAGGATTTC
R333D_forward	CCTAGCGATCGCTGATCGCATGGCCGATG
R333D_reverse	CATCGGCCATGCGATCAGCGATCGCTAGG
